# The 9aaTAD Transactivation Domains: From Gal4 to p53

**DOI:** 10.1371/journal.pone.0162842

**Published:** 2016-09-12

**Authors:** Martin Piskacek, Marek Havelka, Martina Rezacova, Andrea Knight

**Affiliations:** 1 Laboratory of Cancer Biology and Genetics, Department of Pathological Physiology, Faculty of Medicine, Masaryk University Brno, Kamenice 5, 625 00, Brno, Czech Republic; 2 Gamma Delta T Cell Laboratory, Department of Pathological Physiology, Faculty of Medicine, Masaryk University Brno, Kamenice 5, 625 00, Brno, Czech Republic; Virginia Commonwealth University, UNITED STATES

## Abstract

The family of the Nine amino acid Transactivation Domain, 9aaTAD family, comprises currently over 40 members. The 9aaTAD domains are universally recognized by the transcriptional machinery from yeast to man. We had identified the 9aaTAD domains in the p53, Msn2, Pdr1 and B42 activators by our prediction algorithm. In this study, their competence to activate transcription as small peptides was proven. Not surprisingly, we elicited immense 9aaTAD divergence in hundreds of identified orthologs and numerous examples of the 9aaTAD species' convergence. We found unforeseen similarity of the mammalian p53 with yeast Gal4 9aaTAD domains. Furthermore, we identified artificial 9aaTAD domains generated accidentally by others. From an evolutionary perspective, the observed easiness to generate 9aaTAD transactivation domains indicates the natural advantage for spontaneous generation of transcription factors from DNA binding precursors.

## Introduction

The transcription factors are versatile regulators of gene expression. Their DNA binding domains, DBD, specifically recognize regulatory elements and their transactivation domains, TAD, mediate activation of transcription. A number of tested TADs is functional in both yeast and mammals e.g. Gal4 and p53 transcription factors [1,2].

The Nine amino acid Transactivation Domain, 9aaTAD, is universally recognized by the transcriptional machinery in eukaryotes. Currently, the 9aaTAD family comprises of over 40 members including Gal4, Oaf1, Pip2, Pdr1, Pdr3, Leu3, Tea1, Pho4, Gln3, Gcn4, Msn2, Msn4, Rtg3, E2A, MLL, p53-TADI, p53-TADII, FOXO3, NF-kB, NFAT, CEBPA/E, ESX, ELF3, ETV1, KLF2/4, EBNA2, VP16, HSF1, HSF2, HsfA, Gli3, Sox18, PIF, Dreb2a, MTF1, OREB1, WRKY45, NS1, MKL1, VP16, EBNA2, KBP220, ECapLL, P201, AH, and B42 transcription factors. We and others have shown the 9aaTAD domains have competence to activate transcription as small peptides [3–17]. We have established the 9aaTAD prediction service online (www.piskacek.org). The 9aaTADs are annotated on protein database UniProt (www.uniprot.org/9aaTAD).

Previously, we predicted two distinct 9aaTAD domains for p53 protein with conserved proximal Leucines [4,16]. Both 9aaTAD domains, called the 9aaTAD-I and the 9aaTAD-II corresponded with the transactivation regions interacting with the KIX domain of CBP [2,18,19]. We have reported that the first transactivation domain of the p53 protein has the highest similarity to the 9aaTAD of the transcription factor E2A (helical structure of the 9aaTAD-I about 12 aa), while the second transactivation domain of p53 has the highest similarity to the 9aaTAD of transcription factor MLL (shorter helical structure of the 9aaTAD-II about 9 aa) [16].

In this study we aimed to determine the evolutional conservation of the 9aaTAD domains and to prove their competence to activate transcription.

## Materials and Methods

### Constructs

The construct pBTM116-HA (BHA) was generated by Klenow fill-in of oligonucleotides and subcloned in to pBTM116 (B) EcoRI. G1-G45 and H1-H45 were generated by PCR and subcloned in to pBTM116 EcoRI and BamHI sites. All constructs were sequenced by Eurofins Genomics. All construct information, primer sequences and further detailed information are available on request.

### Assessment of enzyme activities

β-galactosidase activity was determined in the yeast strain L40 crude extracts using the ONPG substrate [3]. The average value of β-galactosidase activities from at least three experiments is presented as a percentage with standard deviation (means and plusmn; SD; n = 3).

### Protein purification

The GST-KIX expression constructs kindly provided by Isabelle Lemasson [20] were then transformed into *Escherichia coli* BL21. Cells were grown in LB medium at 37°C and induced with 1 mM IPTG for 2 h at 25°C. The cells were harvested by centrifugation, suspended in lysis buffer with complete protease inhibitors EDTA-free (Roche 04719948001). Protein extracts were cleared by centrifugation for 10 min at 12,000 g, diluted 10 times with GST buffer (50 mM Tris-HCl pH 6.8, 200 mM NaCl, 5% Glycerol), applied on 20 uL GST beads slurry, incubated for 10 min with gently inverting and washed 4 times. Protein purity was estimated by SDS-PAGE. The yeast strain L40 crude extracts were produced by lysis (lysis buffer Roche 04719948001 with complete protease inhibitors EDTA-free) for 15 min at 25°C and clarified by centrifugation 14.000g for 10 min at 4°C.

## Results

### The 9aaTADs in the p53 protein

For both predicted p53 9aaTAD domains, we aimed to prove their competence to activate transcription.

**Fig 1:**
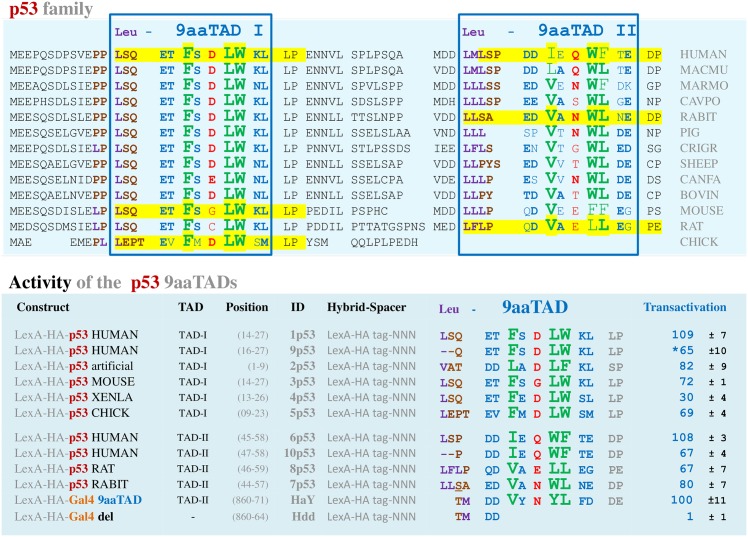
Predicted 9aaTADs in p53 family. Alignment of predicted 9aaTADs in p53 family retrieved by our 9aaTAD prediction algorithms. Highlighted domains were tested for competence to activate transcription in this study.

The human p53 9aaTAD constructs were generated with and without conserved 9aaTAD proximal regions including Leucines. We generated p53 9aaTAD constructs from several other species representing variability within the family (mouse, frog and chicken for TAD-I, and rat and rabbit for TAD-II; highlighted in Fig 1).

**Fig 2:**
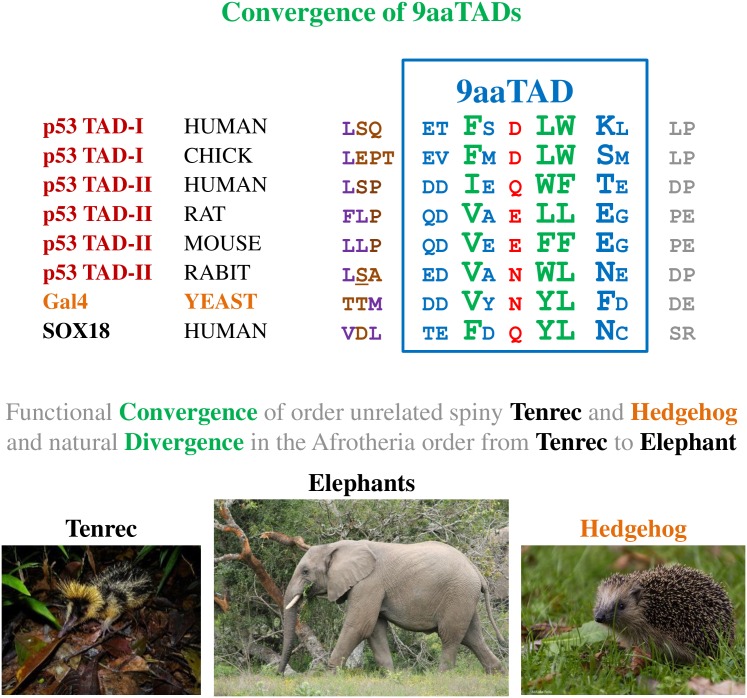
p53 9aaTADs activate transcription as small peptides. The predicted 9aaTADs in p53 from different species were tested for activation of transcription in LexA hybrid constructs. Similarity of p53 with Gal4 and Sox18 are highlighted. The construct 9p53, labelled with asterisk, has lower expression level compared with other constructs (S1 Fig). Animal picture from Flickr: Lowland Streaked Tenrec, Mantadia, Madagascar, Author: Frank Vassen; Elephant, Author: Jon Mountjoy; Igel (Hedgehog), Author: Mi chaela. All pictures have Creative Commons Attribution 2.0 Generic license.

Importantly, we observed the distinctive similarities of the p53 9aaTAD-II domain with Gal4 (four out of nine amino acids are identical and seven out of nine are similar between rabbit p53 and Gal4 9aaTAD)(Fig 1). The fact that the Gal4 proteins could be found only in lower eukaryotes while the p53 proteins are found only in higher eukaryotes, we argue that their 9aaTAD domains' similarity reflects rather functional convergence than conservation (Fig 2). As convergence could be seen in nature e.g. tenrec is genetically closer relative to elephant but rather distant to hedgehog, then tenrec and hedgehog are much more similar by appearance, size and way of functional "spiny" protection (Fig 2).

**Fig 3:**
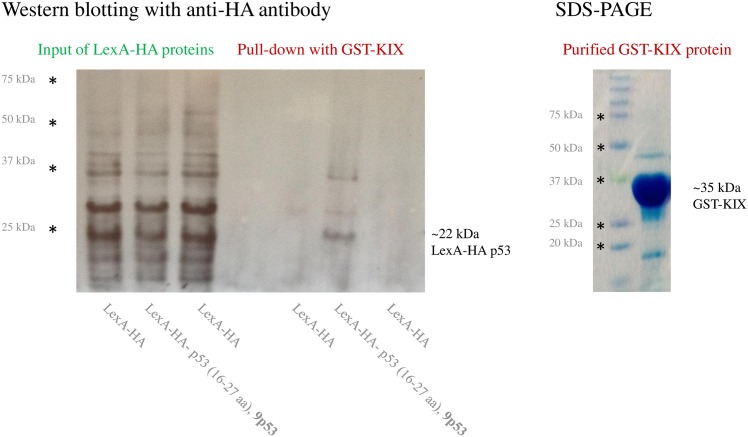
p53 9aaTAD-I is sufficient for interaction with the KIX domain. Interaction of the KIX domain with the p53 9aaTAD-I without its proximal Leucine in pull down experiment was monitored by western blotting. The purity of GST-KIX protein was monitored by SDS-PAGE.

Next, we tested minimal p53 9aaTAD-I domain for interaction with the KIX domain. The LexA hybrid construct with HA-tag and minimal p53 9aaTAD-I domain was expressed in yeast L40 strain. The GST construct with human KIX domain was expressed separately in E.coli strain BL21. Under our experimental conditions, the purified GST-KIX protein was able to specifically pull down p53 9aaTAD-I from whole crude yeast extract (Fig 3).

Finally, we also generated an artificial derivate of the human p53 9aaTAD-I, in which we followed variation found in the p53 TAD-II domain and substituted conserved Tryptophan in TAD-I domain for Phenylalanine conserved in TAD-II domain.

All tested p53 9aaTAD domains have competence to activate transcription (Fig 1). The protein expression of all p53 constructs were confirmed by Western blotting (S1 Fig).

### The 9aaTAD domain and MED15

The transcription factors including Gal4, Oaf1 and Pdr1, members of the 9aaTAD family, are known to interact with the MED15 transcriptional mediator. Therefore we focused our attention on other MED15 interacting proteins such as Msn2 and Msn4 transcription factors [21, 22]. The authors successfully used computational prediction for unstructured regions of the Msn2 protein to localize the transactivation domain.

**Fig 4:**
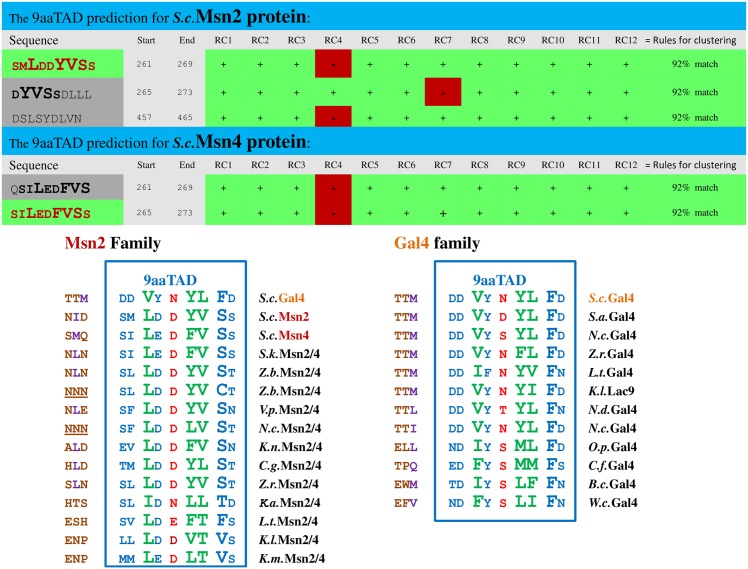
Predicted 9aaTADs in Msn2 family. Prediction result for 9aaTADs in Msn2 and Msn4 activators retrieved by our 9aaTAD prediction algorithms. The conservation and variability of the 9aaTADs in the Msn2 and Gal4 families are shown.

Initially, we used the ExPASy SIB BLAST to identify the Msn2 and Msn4 orthologs. By using our online 9aaTAD prediction, we have identified the 9aaTAD domains in Msn2, Msn4 and their orthologs. First, we showed that the predicted 9aaTADs were located within the reported transactivation domains (Fig 4). Second, we observed conservation in the 9aaTAD domain in both Msn2 and Gal4 families (Fig 4). Next, we chose representative members of the Msn2 family and tested their predicted 9aaTAD domains with and without 9aaTAD proximal regions for ability to activate transcription.

**Fig 5:**
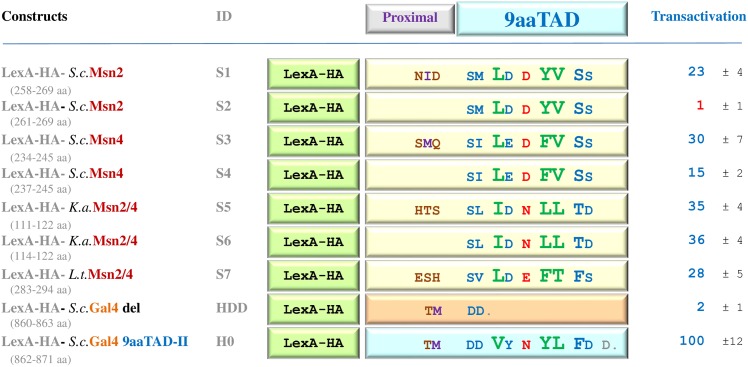
Activity of Msn2 and Msn4 9aaTADs. Msn2 and Msn4 LexA hybrid constructs assayed in L40 strain for transactivation activity.

The resulted activity of all tested Msn2 and Msn4 9aaTAD domains with proximal regions showed up to 30% activity of the Gal4 9aaTAD in LexA hybrid assay, which proved their competence to efficiently activate transcription. The 9aaTAD proximal regions are essential in *S*.*c*.Msn2 and *S*.*c*.Msn4 but not in *K*.*a*.Msn2/4 (Fig 5). Therefore, we assigned both Msn2 and Msn4 transcription factors as proved members of the 9aaTAD family.

Our previously reported 9aaTAD domains for Gal4, Oaf1, Pdr1, Pdr3, Pip2 and other Gal4 paralogs (description and 9aaTAD online annotations, 2006) [4,7] had encouraged further studies with Oaf1 and Pdr1 9aaTAD peptides [23,24]. A novel xenobiotic response domain was reported for Oaf1 and Pdr1 activators and each interacted with the KIX domain of Med15 [23,24]. However, the origin of the Pdr1 peptide (about twelve amino acids) used in the study was unknown (the peptide has not been referred to any screen or prediction e.g. for xenobiotic response pattern).

**Fig 6:**
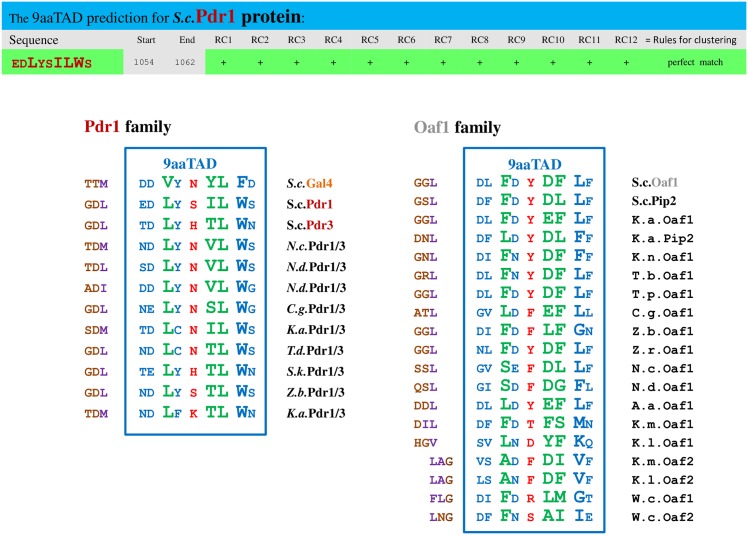
Predicted 9aaTADs in Gal4 family. Prediction results for 9aaTADs in Pdr1 and Pdr3 activators revealed by our 9aaTAD prediction algorithms. The conservation and variability of the 9aaTADs in the Pdr1 and Oaf1 families are shown.

**Fig 7:**
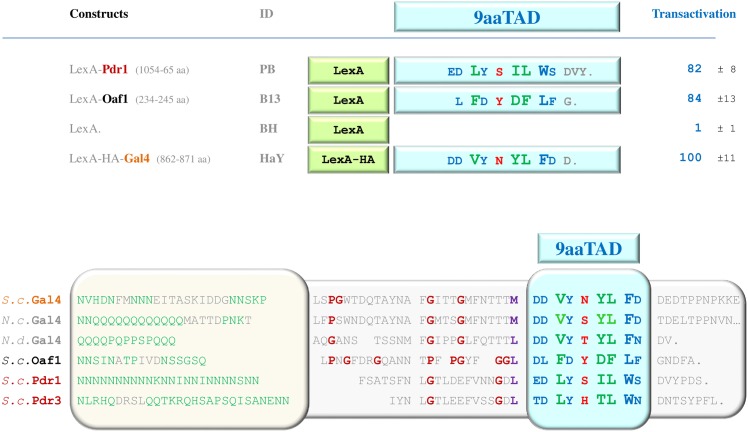
Activity of Pdr1 9aaTAD. Pdr1 LexA hybrid construct assayed in L40 strain for transactivation activity. The similarity with other Gal4 orthologs with adjacent sequences is shown.

We used the ExPASy SIB BLAST to identify the Pdr1 and Pdr3 orthologs and confirmed the 9aaTAD conservation in the family (Fig 6). Because of the position of predicted Pdr1 9aaTAD domain and the Pdr1 xenobiotic domain were matched, we aimed to prove the corresponding Pdr1 peptide for competence to activate transcription. We have tested the Pdr1 xenobiotic domain (12 aa) including predicted 9aaTAD in LexA hybrid assay for activation of transcription. The resulted activity was comparable with the Gal4 and Oaf1 9aaTADs (Fig 7). Therefore, the Pdr1 xenobiotic domain is a functional transactivation domain conserved in Pdr1 family and is identical to reported Pdr1 9aaTAD domain. Therefore, we also assigned Pdr1 as proved member of the 9aaTAD family.

### Spontaneously generated 9aaTADs in Gal4

In recent study (Piskacek et al., 2016), we reported artificial 9aaTAD domains identified by online 9aaTAD prediction (www.piskacek.org) in Gal4 TAD replicas G80BP-A and G80BP-B originally shown in [25]. Similarly, we identified artificial 9aaTAD domain in a strong activator KBP2.20 and in p53 mimetic ECapLL [16][26–29]. Accordingly, we generated an artificial Gcn4 mimetic S11, artificial 9aaTAD domain swapping of the Gal4 residues in to the Gcn4 9aaTAD domain shown in (S4 Fig).

**Fig 8:**
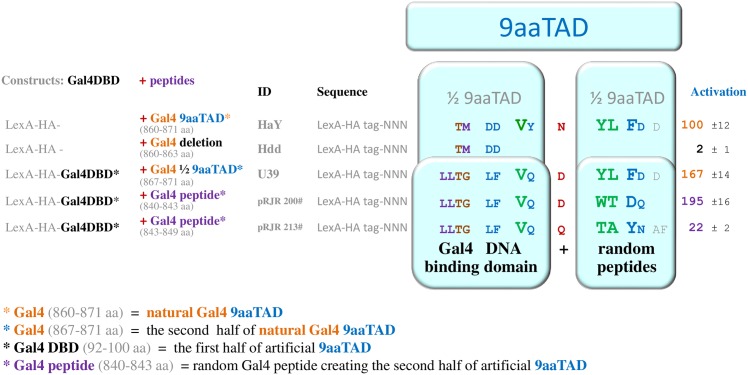
C-terminus of the Gal4DBD domain (92–100 aa) works as a half side of the 9aaTAD domain. A serious concern was found for not real activation function of the Gal4 acidic domain. The artificial 9aaTADs in pRJR200 and pRJR213 constructs were generated accidentally by others and represent so called Gal4 acidic domain. Here we restricted essential part of the Gal4 acidic domain to the recognized functional 9aaTAD region. In this constructs, the functionally unrelated peptides from Gal4 region (840–857 aa) subsidised unintentionally for the second half site of 9aaTAD domain. Artificial 9aaTADs was generated by fusion of the Gal4DBD domain and a half of the 9aaTAD of Gal4. The part of the Gal4DBD domain (92–100 aa) represent first half site of the 9aaTAD domain and was use in constructs to demonstrate capability to generate artificial 9aaTADs by fusion with the second half site of the Gal4 9aaTAD domain.

We predicted a half site of the 9aaTAD domain in Gal4 region (92–100 aa), a part of DNA binding domain, DBD, which is not involved in transactivation. This Gal4 region can be fused with other peptides to form strong artificial 9aaTAD domains. We demonstrated the ability of the Gal4DBD to form strong artificial 9aaTAD domain by fusing it with the second half site of the Gal4 9aaTAD domain (construct U39, Fig 8). The fusion construct of Gal4DBD and a half site of the Gal4 9aaTAD domain activated transcription much powerfully than the natural Gal4 9aaTAD domain.

The best examples of accidentally generated artificial 9aaTAD domains involving Gal4DBD domain are the constructs pRJR200 and pRJR213 generated in [30]. In these constructs, the Gal4DBD domains were fused with peptides originated from Gal4 region (840–857 aa), which resulted in formation of strong artificial 9aaTAD called by authors the Gal4 acidic domain. The Gal4DBD fusion constructs accidentally generated artificial 9aaTADs with strong activities (four and six amino acid long peptides of the artificial Gal4 acidic domain) (Fig 8). For a complete set of Gal4 constructs and their natural activity see recent Piskacek et al., 2006.

Similarly, as indicated by alanine scanning for essential amino acids, another fusion protein Gal4DBD-P201 [31] formed artificial 9aaTAD domain shown (S2 Fig). Furthermore, the Gal4DBD-P201 construct strongly resembles Gal4DBD+1/2 Gal4TAD construct that we described above (Fig 8).

The diverse synthetic peptide libraries revealed strong artificially transactivation domains; "Activating regions: as many as you like" [32]. Beside the proline and tryptophan repeats, we could identify artificial 9aaTAD domains in the reported activators, e.g. the most potent activator reported called B42 [33]. We tested the B42 9aaTAD domain in LexA hybrid assay for activation of transcription. The resulted activity was comparable with the Gal4 9aaTAD domain (S3 Fig). We obtained similar prediction result (predicted 9aaTAD domain: DTLYLDWLED) for other potent activator B114 that has been reported later (in second series of potent activators) [34].

From the results above, it apparent that spontaneous generation of numerous artificial transactivation domains could be generated by intension [25–29] or by accident [30,32–35]. The artificial activators G80BP-A and G80BP-B (Gal4 9aaTAD mimetics)[25], KBP2.20 (KIX binding peptide, random peptide from screen)[26], ECapLL (p53 derivate)[27–29], S11 (Gcn4 / Gal4 derivate, 9aaTAD domain swapping derivate) (S4 Fig), pRJR200 and pRJR213 (Gal4 acidic domain constructs, artificial 9aaTAD generated accidentally) [30], U39 (Gal4 derivate, artificial 9aaTAD made by fusion, analogy to pRJR200 and pRJR213, generated intentionally)(Fig 8) and B42 (artificial 9aaTAD, random peptide from screening) (S3 Fig) activators were assigned to 9aaTAD family.

## Discussion

**Fig 9:**
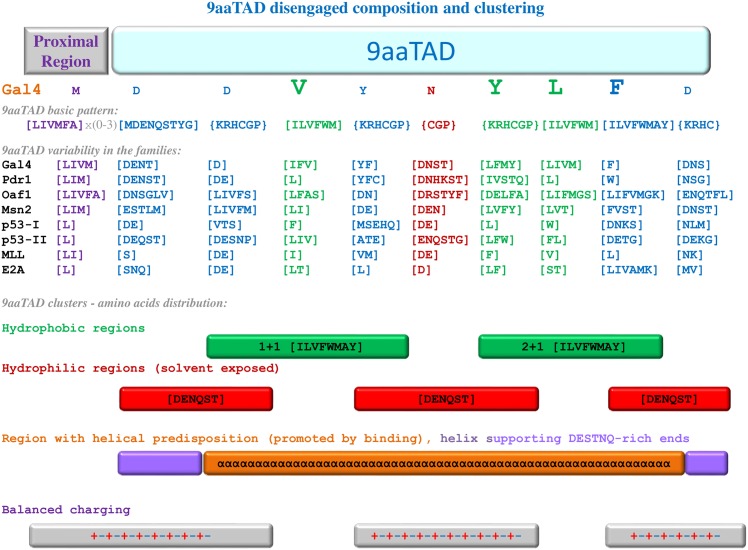
The 9aaTAD domain disengaged composition and clustering.

The Nine amino acid Transactivation Domain, 9aaTAD, is a large family of the transcription activators universally recognized by transcriptional machinery from yeast to man. The 9aaTAD domain is characterized by the disengaged pattern, by amino acid composition and by tandem of hydrophobic clusters (Fig 9). The 9aaTAD domain is well balanced by hydrophilic amino acids, which are usually in proportion of positively and negatively charged. From the structural data for the E2A and MLL in complex with the KIX domain, we observed helix formation for some 9aaTADs, whose length vary from 9 to 12 aa [16]. The online 9aaTAD prediction is available on www.piskacek.org.

Some but not all of the 9aaTAD domains interact with multiple mediators and that with different binding affinity e.g. p53, MLL and E2A (MLL-site and Myb-site of KIX domain, sites of TAZ1 and TAZ2, and IBiD) [23,36–40]. These interactions share some similarity, but also show obvious differences and individuality, e.g. Oaf1 and Pdr1 [23,24]. The 9aaTAD domains may use multiple binding positions and orientations, e.g. p53 and Gcn4 [19,41].

Albeit the 9aaTAD domains have enormous variability and amorphous character, they are universally recognized by transcriptional machinery throughout eukaryotes [1]. The 9aaTAD transcription factors interact with multiple transcriptional mediators [18,19,23,24,26,29,42–54][55–60]. The conservation of some transcriptional mediators (TAF9 and KIX domain in MED15) might be responsible for the 9aaTAD domain overall occurrence and functionality.

In this study, we showed that both p53 9aaTAD domains activate transcription as small peptides. Nevertheless, we showed that the p53 9aaTAD-I and 9aaTAD-II domains do not have one amino acid identical. We also demonstrated that their shared 9aaTAD motif enables residue swapping. In respect of the shared 9aaTAD motif in p53 protein, the modification of the first p53 9aaTAD-I domain towards the second 9aaTAD motif (in the p53 9aaTAD-II domain) did not result in a lost of the transactivation potential (artificial construct 2p53).

Similar result has been reported for p53 mimetic ECapLL [28]. We recognized analogical modification of the second p53 9aaTAD-II domain towards the first 9aaTAD motif (in the p53 9aaTAD-I domain) [16]. Noteworthy, both p53 9aaTAD-I and 9aaTAD-II domains bind to the same transcriptional mediator subunits of the CBP/p300, what further underpins the shared 9aaTAD motif (MLL-site and Myb-site of KIX domain, sites of TAZ1 and TAZ2, and IBiD)[19,36].

Our results have shown the 9aaTAD convergence in the rabbit p53 and Gal4 proteins, which have 44% overall identity and 78% similarity. Both 9aaTAD domains resembled another 9aaTAD domain, Sox18 [61]. Previously, we observed another 9aaTAD convergence in two unrelated proteins, E2A and MLL (SDLL-D-FS and SDIM-D-FV). Their 9aaTAD domains occupied identical binding site on the KIX domain of the CBP [16]. However, we found only negligible similarity between rat and human p53 TAD-II, which represented natural 9aaTAD divergence.

The 9aaTAD proximal Leucines are well conserved in the p53 family, but less in other members of the 9aaTAD family, e.g. Msn2 activators. The Leucines are not conserved also in other 9aaTAD proximal regions demonstrating overall natural variability (Isoleucine, Valine, Phenylalanine, Methionine, Threonine and Serine were found in the 9aaTAD proximal regions of MLL, Oaf1, Pdr1, Gcn4 and Gal4). From our results for the Msn2 and p53 activators, we demonstrated that the contribution of the 9aaTAD proximal regions is individually variable and not always essential for transactivation function. The 9aaTAD proximal regions might be part of the 9aaTAD helix e.g. E2A and MLL [16].

The members of the 9aaTAD family share small size, highly variable pattern and hydrophobic and hydrophilic clusters. The 9aaTAD domains could be split in two subdomains and merged with other 9aaTAD subdomains without losing activity. The 9aaTAD domains could be largely and almost freely modified, and new 9aaTAD domain could be easily generated by accident or by intention.

The 9aaTAD family is not exclusive transactivation domain in eukaryotes. There are numerous known transactivation domains unrelated to the 9aaTAD domain with different mode of binding to transcriptional mediators e.g. STAT2 [62] or different amino acid composition e.g. SP1 [63–65].

During the evolution, the fast generation of numerous specific transcription factors has been crucial for tailored regulation of individual genes. The observed easiness of spontaneous generation of artificial 9aaTAD transactivation domains in the labs, "Activating regions: as many as you like" [32], indicates that the 9aaTAD domain represented evolutional advantage for generation of transcription factors from DNA binding precursors.

## Supporting Information

S1 Fig Protein expression. The protein level produced from the constructs 1-10p53, HaA and HaY in L40 strain were monitored by Westernblotting. The proteins comprise LexA a HA tags with a total size of about 21 kDa. The degradation product comprising almost LexA protein (LexA torso) has a total size of about 20kDa.
(TIF)

S2 Fig Artificial activator P201. We fused the part of the Gal4 DNA binding domain (92–100 aa), DBD, with the second half site of the Gal4 9aaTAD. The Gal4 DNA binding domain region (92–100 aa) substitute for the first half of the 9aaTAD in this and other artificial constructs. The amino acids in fusion region of both Gal4 DNA binding domain and the random peptide are essential for transactivation function. Notice: Gal4 region (1–84 aa) is sufficient for DNA binding. Blue asterisks referred to the results of this study (Fig 8), constructs HaY and U39, black asterisks to the results reported by Lu X et al. 2000 and red asterisks by Lu Z et al. 2005.
(TIF)

S3 Fig Artificial activator B42. The full sequence of B42 peptide (1–79 aa) and the identified 9aaTAD within are shown. The B42 9aaTAD LexA hybrid construct was assayed in L40 strain for the transactivation activity.
(TIF)

S4 Fig Putative 9aaTAD motifs in Gcn4. We identified the putative 9aaTADs in both reported Gcn4 transactivation domains (description and 9aaTAD online annotations, 2006), which amino acid variations are very close to mouse and bovine p53 9aaTADs (5 identical and 3 similar amino acids: **K/Q**, **D/E**, **V**, **E**, **S/T, F, F, D,** N/E). Noteworthy, the Gcn4 protein has an unusual Lys in the position 1 of the 9aaTAD-II, which is out of predictive recognition (sequence: KEWTSLFDN). The unusual amino acids in the 9aaTAD domains were found also in other members of the 9aaTAD family e.g. Cysteine and Glycine in rat and mouse p53 9aaTAD-I. We assigned many transactivation domains to the 9aaTAD family, which fit with size, share deliberated 9aaTAD pattern and the clusters of the hydrophobic/hydrophilic amino acids. The amorphous nature of the 9aaTAD domains does not offer any invariant or conserved residues, which let us to generate the absolute reliable pattern for all of them. Therefore i) our prediction is still uncertain, ii) generate many false positives, iii) pattern does not fit for all 9aaTAD variations of the orthologs, and iv) putative 9aaTADs need always to be experimental verified. Nevertheless, there are many examples, where the 9aaTAD prediction works well, e.g. MLL or p53 activators. MLL (Q03164) is 3969 amino acids long protein with only two predicted 9aaTADs, where one of them is confirmed transactivation domain. Over two hundred Gcn4 9aaTAD-I modifications were generated and their competence to activate transcription were assayed by Warfied et al., 2014. Despite of the authors' enormous effort to define the transactivation domain by this approach, they found merely Tryptophan-rich transactivation domains deprived of acidic residues (AVWWSLFAS, AWWWWAFWS, AFWMWLFAT). We tested the Tryptophan-rich activation domain m120 (AFWMWLFAT) derived from Gcn4 9aaTAD in the standard LexA hybrid assay. The Gcn4 mutant m120 has no activity (>1% ± 1 of the referent Gal4 construct HaY), what indicated serious data inconsistency in the report by Warfied et al., 2014. Therefore we proceeded differently to characterise the Gcn4 TAD. Because of the Gcn4 TAD-I domain fulfils the deliberate criteria for 9aaTAD motif (positive online 9aaTAD prediction, formation of two hydrophobic patches interspersed by hydrophilic residues), we made subdomain swapping between putative Gcn4 9aaTAD and Gal4 9aaTAD and generated a hybrid construct S11 to prove predicted shared motif. The construct S11 has comparable transcriptional activity to the Gal4 9aaTAD in LexA hybrid assay, what proofs the concept for the 9aaTAD motif in Gcn4 TAD-I by the swapping experiment (Gcn4 9aaTAD core spreading from position 3p to 7p was swapped with Gal4 9aaTAD). Noteworthy, the hydrophobic patches in the core of Gcn4 9aaTAD-I (**V**_**SFF**) and Gcn4 9aaTAD-II (w_**SLF**) have high similarity with the 9aaTAD domain of the *B*.*a*.Gal4 (I_**SLF**).
(TIF)
