# Supplementary figures and images for: The 9aaTAD Transactivation Domains: From Gal4 to p53
Source: PLoS One. 2016 Sep 12;11(9):e0162842. doi: 10.1371/journal.pone.0162842 (PMC5019370; doi:10.1371/journal.pone.0162842)

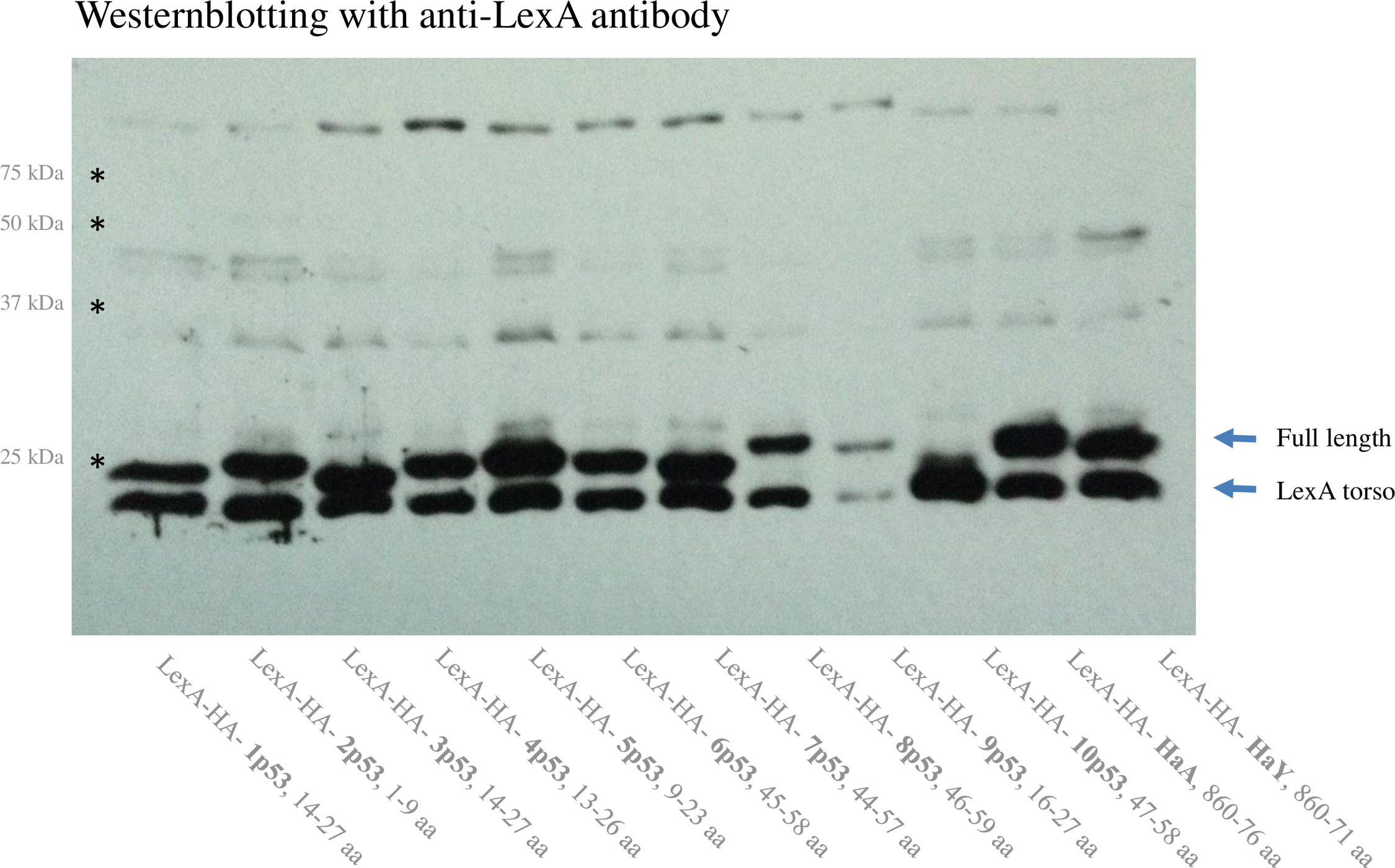

Supplement: S1 Fig — The protein level produced from the constructs 1-10p53, HaA and HaY in L40 strain were monitored by Westernblotting. The proteins comprise LexA a HA tags with a total size of about 21 kDa. The degradation product comprising almost LexA protein (LexA torso) has a total size of about 20kDa. (TIF) [file pone.0162842.s001.tif]

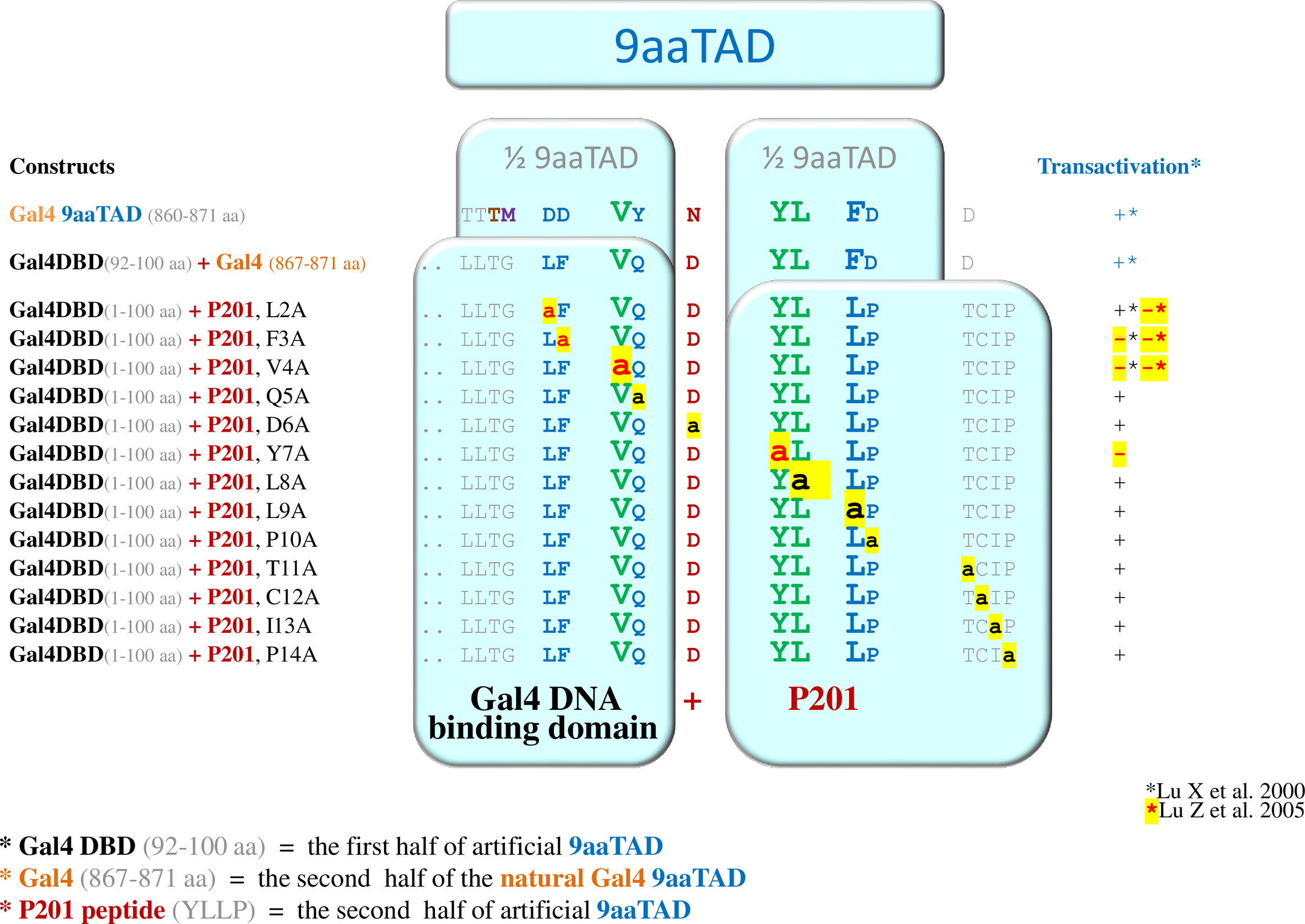

Supplement: S2 Fig — We fused the part of the Gal4 DNA binding domain (92–100 aa), DBD, with the second half site of the Gal4 9aaTAD. The Gal4 DNA binding domain region (92–100 aa) substitute for the first half of the 9aaTAD in this and other artificial constructs. The amino acids in fusion region of both Gal4 DNA binding domain and the random peptide are essential for transactivation function. Notice: Gal4 region (1–84 aa) is sufficient for DNA binding. Blue asterisks referred to the results of this study (Fig 8), constructs HaY and U39, black asterisks to the results reported by Lu X et al. 2000 and red asterisks by Lu Z et al. 2005. (TIF) [file pone.0162842.s002.tif]

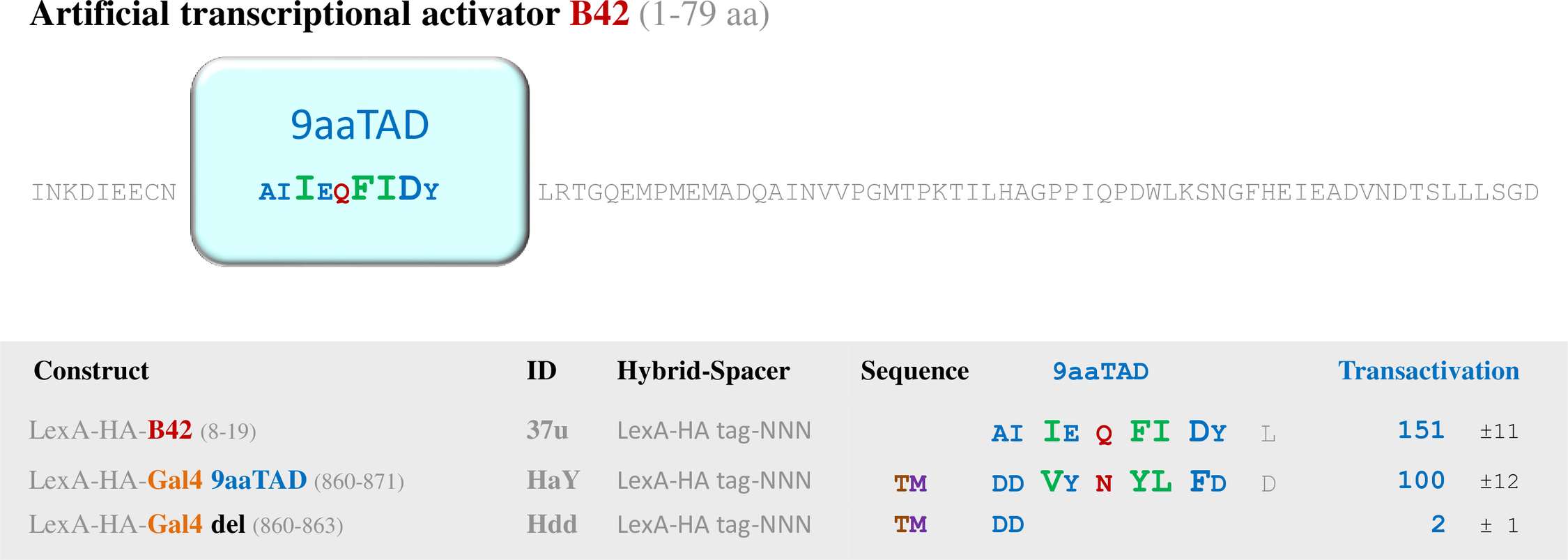

Supplement: S3 Fig — The full sequence of B42 peptide (1–79 aa) and the identified 9aaTAD within are shown. The B42 9aaTAD LexA hybrid construct was assayed in L40 strain for the transactivation activity. (TIF) [file pone.0162842.s003.tif]

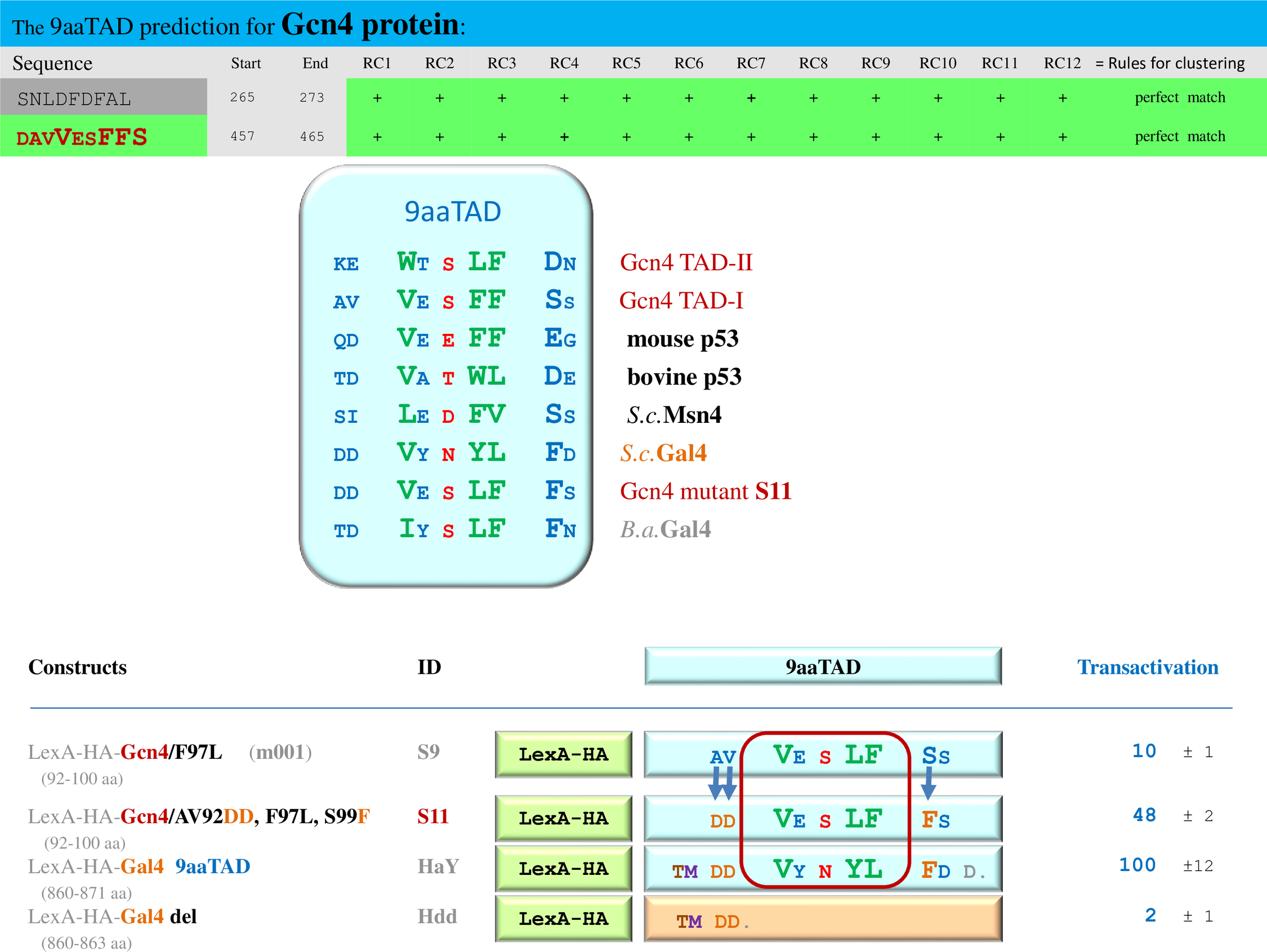

Supplement: S4 Fig — We identified the putative 9aaTADs in both reported Gcn4 transactivation domains (description and 9aaTAD online annotations, 2006), which amino acid variations are very close to mouse and bovine p53 9aaTADs (5 identical and 3 similar amino acids: K/Q, D/E, V, E, S/T, F, F, D, N/E). Noteworthy, the Gcn4 protein has an unusual Lys in the position 1 of the 9aaTAD-II, which is out of predictive recognition (sequence: KEWTSLFDN). The unusual amino acids in the 9aaTAD domains were found also in other members of the 9aaTAD family e.g. Cysteine and Glycine in rat and mouse p53 9aaTAD-I. We assigned many transactivation domains to the 9aaTAD family, which fit with size, share deliberated 9aaTAD pattern and the clusters of the hydrophobic/hydrophilic amino acids. The amorphous nature of the 9aaTAD domains does not offer any invariant or conserved residues, which let us to generate the absolute reliable pattern for all of them. Therefore i) our prediction is still uncertain, ii) generate many false positives, iii) pattern does not fit for all 9aaTAD variations of the orthologs, and iv) putative 9aaTADs need always to be experimental verified. Nevertheless, there are many examples, where the 9aaTAD prediction works well, e.g. MLL or p53 activators. MLL (Q03164) is 3969 amino acids long protein with only two predicted 9aaTADs, where one of them is confirmed transactivation domain. Over two hundred Gcn4 9aaTAD-I modifications were generated and their competence to activate transcription were assayed by Warfied et al., 2014. Despite of the authors' enormous effort to define the transactivation domain by this approach, they found merely Tryptophan-rich transactivation domains deprived of acidic residues (AVWWSLFAS, AWWWWAFWS, AFWMWLFAT). We tested the Tryptophan-rich activation domain m120 (AFWMWLFAT) derived from Gcn4 9aaTAD in the standard LexA hybrid assay. The Gcn4 mutant m120 has no activity (>1% ± 1 of the referent Gal4 construct HaY), what indicated serious data inconsistenc [file pone.0162842.s004.tif]
